# Multi-Objective Optimization for Grinding Parameters of 20CrMnTiH Gear with Ceramic Microcrystalline Corundum

**DOI:** 10.3390/ma12081352

**Published:** 2019-04-25

**Authors:** Shengyong Zhang, Genbao Zhang, Yan Ran, Zhichao Wang, Wen Wang

**Affiliations:** Key State Laboratory of Mechanical Transmission, Chongqing University, Chongqing 400044, China; rainbowzhang269@163.com (S.Z.); gen.bao.zhang@263.net (G.Z.); freedomoverman@hotmail.com (Z.W.); wangwen_cqu@163.com (W.W.)

**Keywords:** ceramic microcrystalline corundum, 20CrMnTiH, pixel method, grinding parameters, grinding indicators, multi-objective optimization

## Abstract

(1) The alloy material 20CrMnTiH is widely used in gear manufacturing, but difficult to process, and its quantity (efficiency) and quality (surface quality) are generally negative correlation indicators. As a difficult but realistic problem, it is of important practical significance to explore how to efficiently grind high-precision low-carbon alloy gear workpieces. (2) Firstly, the pixel method was applied to analyze the grinding principles and explore the grinding parameters—the grinding wheel speed and grinding wheel frame moving speed—as well as the feed rate, which impacts the grinding indicators. Secondly, based on the ceramic microcrystalline corundum grinding wheel and the 20CrMnTiH gear workpiece, controlled experiments with 28 groups of grinding parameters were conducted. Moreover, the impact curves of the grinding parameters on the grinding indicators—the grinding efficiency, grinding wheel life, and surface roughness—were obtained by the multiple linear regression method. Finally, the multi-objective optimization method was used to comprehensively optimize the grinding process. (3) Compared with the traditional grinding process, under optimized grinding parameters, the 20CrMnTiH gear workpieces have a lower surface roughness and a longer grinding wheel life, and require a shorter time to achieve grinding accuracy. (4) The grinding experiments showed that the grinding parameters are linearly related to the grinding indicators. The optimization results show that the precision, efficiency, and economy of the 20CrMnTiH gear grinding process have been improved via the comprehensive optimization of the grinding parameters.

## 1. Introduction

As the second high-hardness material in nature, the microcrystalline corundum has dense texture and sharp-pointed particles, which has a significant and decisive impact on the material’s properties [1]. Ceramics and resins are generally used as its binders for grinding, polishing, sand blasting, and precision casting. Furthermore, the ceramic microcrystalline corundum is more and more applied as the material of the grinding wheel to grind the 20CrMnTiH gear workpiece. The selection of binder and composite abrasive is extraordinarily important, as the abrasive composition has a large influence on the physical properties of the grinding wheel [2,3]. For example, corundum composite abrasives containing Fe–Ce [4,5] and Ti [6] were used to grind diamond films more efficiently. The ceramic microcrystalline corundum grinding wheel prepared by hollow ball corundum particles as a pore former [7] has good microstructure and heat dissipation [8]. A large number of experiments have shown that because of the excellent hardness and heat dissipation characteristics, microcrystalline corundum grinding wheels are applied to many kinds of grinding methods. Wang et al. studied the theoretical analysis and experimental verification of the grinding principle of the new microcrystalline corundum’s forming method [9]. Breitung-Faes et al. studied the true grinding performance of molten corundum [10]; Yang et al. explored the grinding performance of a new microcrystalline corundum grinding wheel for grinding automotive gears [11]. These research studies indicate that the microcrystalline corundum has been more and more applied to precision grinding over the past 10 years, but its grinding performance needs improving. At present, the machine tool companies mainly determine the grinding parameters based on a large amount of processing experience, which causes the grinding process to lack a scientific experimental analysis and theoretical basis.

Due to its high hardness, high wear resistance, good fatigue resistance, and low-temperature impact toughness, 20CrMnTiH is widely used in gears, shafts, and other parts of automobiles and airplanes. However, its high hardness and high wear resistance lead to the difficulty in processing and low grinding efficiency. Therefore, the increasing interest in optimizing the grinding process for better grinding precision performance has heightened the need for more research. Current research on the machining and grinding performance of 20CrMnTiH gears has mainly involved laser, cold forging, milling, and grinding, such as laser surface fatigue resistance [12,13], cold forging surface properties [14,15], and common grinding surface properties [16]. Among them, grinding surface properties are of significance for manufacturing quality, especially assembly performance. In terms of grinding, Wang et al. studied the surface integrity of WD-201 microcrystalline corundum grinding 20CrMnTiH gear workpieces [17], including residual stress, surface roughness, and hardness. Wang et al. evaluated the grinding burn of 20CrMnTiH workpieces based on the binary image and neural network method [18]. Li et al. explored the tribological properties of multilayer graphene and spherical SnAgCu for grinding 20CrMnTiH [19]. Zhang applied the exponential model to optimize the grinding parameters of the cubic boron nitride (CBN) grinding wheel grinding 20CrMnTi [20] with the optimization index of the surface quality. These research studies have mainly focused on obtaining better grinding quality, but have not considered the economic cost or the efficiency. To solve this problem, we comprehensively optimized the grinding process.

In terms of grinding precision, the surface quality of machining includes the surface roughness, surface waviness, work hardening, residual stress, and metallographic changes of the structure, where the work hardening, residual stress [21], and metallographic changes of the structure are the physical mechanics performance of the surface layer. Due to different applications, its quantitative indicators are double-edged [22]. On the other hand, the geometrical features of the surface layer are positively correlated with the grinding quality, wherein the surface roughness is a microscopic geometric feature, and the surface waviness is between microscopic and macroscopic [23]. Furthermore, the surface roughness impacts the mechanical properties, wear resistance, fatigue strength, contact stiffness, vibration, and noise, which are closely related to the service life and reliability of mechanical products. In order to make the surface quality evaluation more accurate, surface roughness Ra is used to characterize the grinding surface quality in this paper.

In terms of grinding efficiency, based on the 20CrMnTiH gear with an outer diameter of 300 mm, this paper distinguishes the grinding time *T_G_* to the accuracy required for rough grinding (Ra: 2 μm), fine grinding (Ra: 1.2 μm), and ultra-fine grinding (Ra: 0.05 μm) as the evaluation index.

In terms of the grinding economy, because the wear of the grinding wheel constantly changes the state of the grinding wheel working surface (Ra: 0.16 to 0.04 μm), the grinding wheel life is selected to present the grinding economics. As the grinding time increases, the cutting ability of the grinding wheel decreases, and various grinding defects—e.g., abrasive passivation, grinding chip clogging, workpiece surface burn—continue to occur, and the surface roughness obtained in the specified grinding time is lower than that which is required. At this point, the grinding process cannot be continued, and the grinding wheel must be dressed. Take precision dressing as an example: one dressing takes 1 h (diameter of grinding wheel: 300 mm, dressing feed rate: 50 mm/min, dressing cutting depth: 0.01 mm, single-side total dressing depth: 0.1 mm). The resultant actual grinding time of the grinding wheel decreases greatly. The grinding wheel life is characterized by the actual grinding time *T_D_* between two dressings in this paper.

The rest of the paper is organized as follows. The materials and methods are introduced in Section 2. The grinding parameters, including the grinding wheel speed, grinding wheel frame moving speed, and feed rate, are obtained via the analysis of the grinding principle in Section 3.1. The group experiments of grinding parameters and grinding indicators are conducted and analyzed in Section 3, including the impact curve. The correlation function for multi-objective optimization is solved in Section 4. The results are discussed in Section 5. The paper is summarized in Section 6.

## 2. Materials and Methods

The grinding combination of ceramic microcrystalline corundum grinding wheels and 20CrMnTiH gear workpieces was selected as the experiment sample for the worm grinding experiment. Twenty-eight sets of comparative control experiments were conducted. The relationship between grinding parameters and indicators is explored. 

### 2.1. Materials

The chemical composition and physical properties of the microcrystalline corundum and 20CrMnTiH are shown in Table 1, Table 2, Table 3 and Table 4.

The main component of microcrystalline corundum is α-Al_2_O_3_, whose hardness is second only to diamond. The ceramic microcrystalline corundum is widely used in grinding due to its excellent toughness, shock resistance, high mechanical strength, and hardness. The 20CrMnTiH has high hardenability, strength, and toughness under the condition of ensuring hardenability, especially high/low temperature impact toughness and good fatigue resistance. However, the difficult grinding process and low grinding efficiency make the optimization of the grinding parameters for the grinding process of great practical significance.

### 2.2. Methods

**Figure 1:**
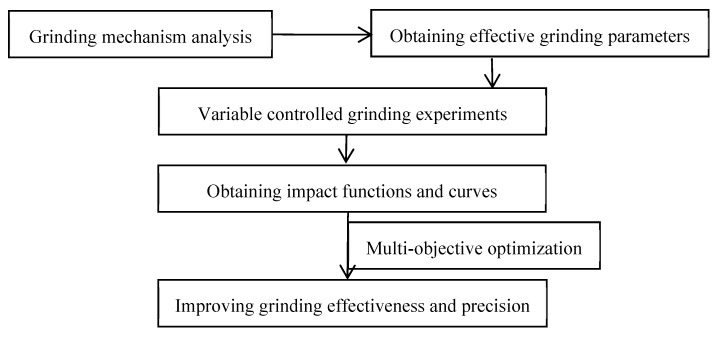
Flow chart of multi-objective optimization for grinding parameters.

**Figure 2:**
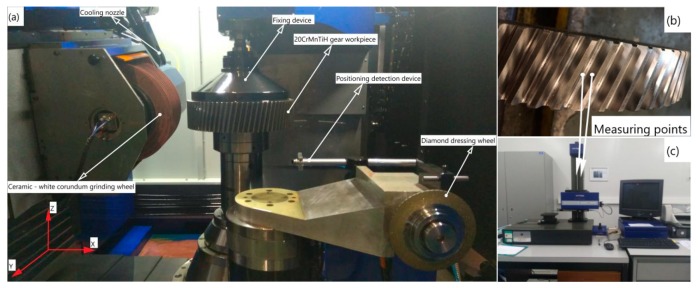
(**a**) Grinding machine; (**b**) Measured points; (**c**) Surface roughness measuring instrument.

The flow chart of the grinding parameter optimization in this paper is shown in Figure 1. Based on the design of the experimental process, 28 sets of comparative control experiments (8, 10, and 10 sets for the feed rate, grinding wheel frame speed, grinding wheel speed, respectively) were conducted on a Chinese grinding machine tool YKZ7230 (Qinchuan Machine Tool & Tool Group Share Co., Ltd., Shaanxi, China). The gear parameters and experiment equipment are shown in Table 5 and Figure 2, respectively.

The experiment data is fitted to obtain the correlation curves between grinding parameters (grinding wheel speed ng, feed rate, *v_h_*; and grinding wheel frame moving speed, *v_v_*) and grinding indicators (surface roughness, Ra; the time required to achieve machining accuracy, *T_G_*; and grinding wheel life, *T_D_*). The multi-objective optimization model of grinding indicators includes Ra, *T_G_*, and *T_D_*. The experiment results show that the optimized grinding parameters can maximize the grinding wheel life and shorten the grinding cycle under the premise of ensuring the processing precision, which confirms the feasibility and superiority of the grinding parameters’ optimization.

## 3. Analysis of Experimental Results

A worm grinding machine is used in this paper. Through the analysis of the grinding process, the grinding parameters affecting the surface roughness, efficiency, and the grinding wheel life are determined. The grouping experiments were carried out according to the determined grinding parameters. 

### 3.1. Worm Grinding Process Analysis

**Figure 3:**
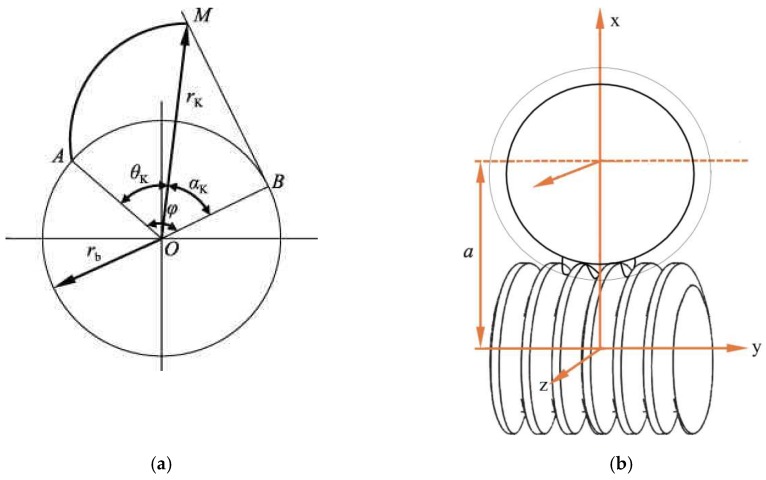
(**a**) Involute forming principle; (**b**) Grinding diagram of worm grinding.

In this paper, the workpiece is an involute helical gear with an outer diameter of 300 mm. The principle of involute formation and the grinding process of the worm grinder are shown in Figure 3.

The pressure angle can be deduced from the involute function $\theta_{K}=inv\alpha_{K}=\tan\alpha_{K}-\alpha_{K}$ as Equation (1).

(1) $$\phi =\theta_{K}+\alpha_{K}=\tan\alpha_{K}$$

**Figure 4:**
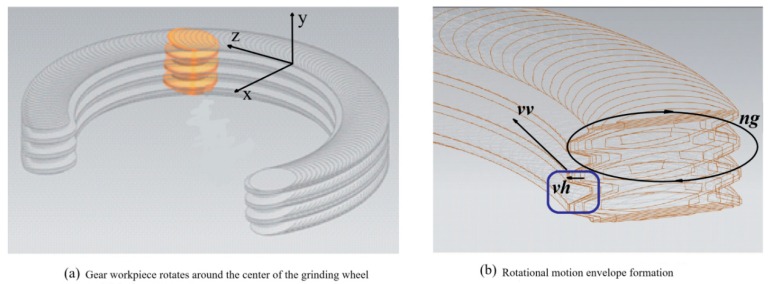
Schematic diagram of the worm wheel grinding principle.

The grinding principle of the worm wheel grinding machine is the same as that of the worm gear grinding machine. Through the relative rotational movement of the gear workpiece and the grinding wheel, the material is cut off under the transverse and longitudinal common feeding, and the tooth surface is formed (Figure 4). 

The envelope curve equation of a point on the grinding wheel in the gear workpiece coordinate system is as shown in Equation (2).

(2) $$r_{g}=[x_{g},y_{g},z_{g}]={\left[\begin{array}{c} x_{gw}\cos\phi -ky_{gw}\sin\phi +v_{h}t\\ kx_{gw}\sin\phi -ky_{gw}\cos\phi \\ z_{gw}+v_{v}t \end{array}\right]}^{T}$$

where *k* = ±1 represents the direction of rotation; (*x_wg_*,* y_wg_*, and *z_wg_*) represent the relative coordinates of the grinding wheel in the workpiece coordinate system, and *Φ* represents the relative rotation angle.

**Figure 5:**
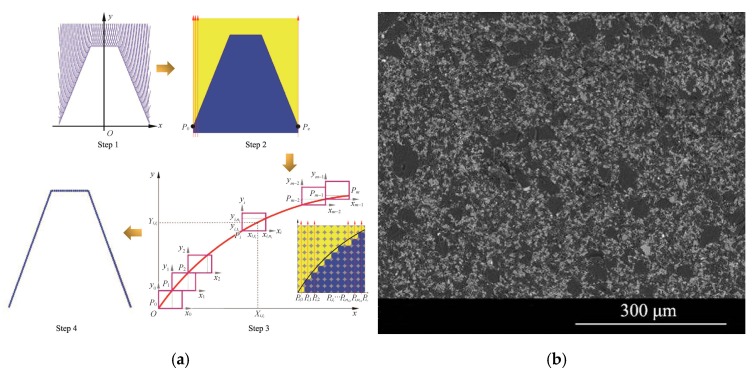
(**a**) The worm wheel grinding principle; (**b**) 20CrMnTiH surface texture.

The grinding effect map is obtained by pixel analysis (Figure 5).

1. Calculating the sweeping surface of the worm enveloping grinding wheel according to the movement relationship between the worm and the forming grinding wheel (step 1);
2. Performing posture enlargement on the swept surface (magnification K1) (step 2);
3. Using the coordinate transformation rule to convert the captured coordinate point set of each interval to a unified coordinate system and obtain a dense data point set that is capable of completely characterizing a complete grinding wheel contour (step 3);
4. Diluting and smoothing the resulting dense data point set to obtain a smooth grinding wheel profile (step 4).

From the analysis of Equation (2), Figure 4b, and Figure 5, in addition to the grinding wheel speed during the grinding process, the transverse direction (feed rate) and the longitudinal feed rate (the wheel frame speed) also have a certain impact on the material removal rate, surface roughness, and grinding wheel life.

### 3.2. Analysis of Grinding Wheel Speed and Efficiency, Grinding Wheel Life, and Surface Roughness

When exploring the relationship between the grinding wheel speed and efficiency, as well as between the grinding wheel life and surface roughness, the grinding wheel speed was controlled as a variable, and the feed rate and grinding wheel frame speed were constant. The grouping processing experiments were carried out according to the commonly used grinding wheel speed interval. The experiment grouping and results are shown in Table 6.

### 3.3. Analysis of Feed Rate and Efficiency, Grinding Wheel Life, and Surface Roughness

The feed rate is a key factor affecting both the surface roughness and wheel life, whose effect is no less than that of the grinding wheel speed. When exploring the relationship between the feed rate and efficiency, as well as between the grinding wheel life and surface roughness, the feed rate was controlled as a variable, and the grinding wheel speed and grinding wheel frame speed were constant. The grouping processing experiment was carried out according to the commonly used feed rate interval. The experiment grouping and results are shown in Table 7.

### 3.4. Analysis of Wheel Frame Speed and Efficiency, Grinding Wheel Life, and Surface Roughness

For exploring the relationship between the grinding wheel frame moving speed and grinding indicators, the grinding wheel frame moving speed was controlled as a variable, and the grinding wheel speed and feed rate were constant. The grouping processing experiment was carried out according to the commonly used grinding wheel frame speed interval (Table 8).

### 3.5. Impact Curve Analysis

**Figure 6:**
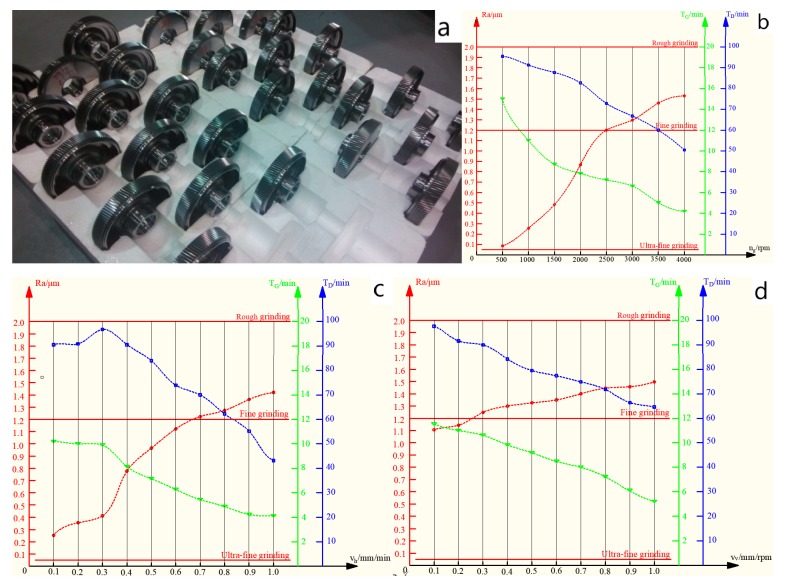
(**a**) Experiment processed gear; analysis of the impact of the (**b**) Grinding wheel speed; (**c**) Feed rate; and (**d**) Grinding wheel frame speed on surface roughness, efficiency, and wheel life.

The ceramic microcrystalline corundum grinding wheel grinding 20CrMnTiH gear workpiece experiment was divided into 28 groups; the processed gear workpiece is shown in Figure 6a. According to the processing data, the impact curves of the grinding parameters on the efficiency, grinding wheel life, and surface roughness were fitted. The fitting results are shown in Figure 6. The results show that due to the influence of resonance, noise, or other external factors, the fitting curve has occasional peak waves, but is linearly correlated as a whole.

## 4. Multi-objective Optimization Results

It can be seen from Figure 6 that when considering a single variable, the grinding parameters are linearly related to the surface roughness, efficiency, and grinding wheel life as a whole, which indicates that the surface roughness, efficiency, and grinding wheel life and are in a multivariate linear relationship with the grinding parameters.

As a branch of mathematical programming, multi-objective optimization aims to study the optimization of multiple targets in a given area. The solution methods mainly include the following:

1. Converting multiple to fewer: converting multi-objects into a single object or double objects that are easy to solve, including the main target method and linear weighting method.
2. The hierarchical sequence method: the targets are sorted according to importance, and each time, the next target optimal solution is obtained in the previous target optimal solution set until the common optimal solution is obtained.
3. The analytic hierarchy method: a combination of qualitative and quantitative methods, which is suitable for fuzzy target variable optimization.

In this paper, the ideal point method is combined with the layered sequence method. Firstly, the impact functions of surface roughness, grinding wheel life, and the time acquired to achieve machining accuracy regarding the grinding parameters are obtained. Moreover, in the solution of meeting the surface roughness requirements, the ideal point method is used to convert the grinding wheel life and the time acquired to achieve machining accuracy into a single target plan, where the weight coefficients of the grinding wheel life $\lambda_{G}$ and the time acquired to achieve machining accuracy $\lambda_{D}$ are the same.

### 4.1. Impact Function Solution

The multivariate linear regression method is used to obtain the multivariate linear functions of surface roughness, efficiency, and grinding wheel life regarding the grinding parameters (Equation (3)):

(3) $$\{\begin{array}{c} Ra=-0.2717+0.0004n_{g}+0.2668v_{h}+0.4739v_{v}\\ T_{G}=24.4720-0.0021n_{g}-7.0533v_{h}-6.1196v_{v}\\ T_{D}=135.2130-0.0080n_{g}-34.4990v_{h}-35.8133v_{v} \end{array}$$

where $500\leq n_{g}\leq 4000$, $0.1\leq T_{G}\leq 1.0$, and $0.1\leq T_{D}\leq 1.0$.

### 4.2. Grinding Parameter Optimization

Obviously, the objective function of grinding parameter optimization cannot guarantee that the surface roughness, efficiency, and grinding wheel life achieve optimization at the same time. In view of the machine tool company having corresponding surface roughness requirements for different grinding stages, the objective function in this paper is to guarantee the difference between the time acquired to achieve grinding accuracy and grinding wheel life optimization under the surface roughness requirement. 

Let $x={\left[n_{g},v_{h},v_{v}\right]}^{T}$. To improve the practical application value of our work, the specified surface roughness of rough grinding (Ra: 2 μm), fine grinding (Ra: 1.2 μm), and ultra-fine grinding (Ra: 0.05 μm) are used as the constraints of multi-objective optimization. The multi-objective programming mathematical model of ceramic microcrystalline corundum grinding 20CrMnTiH is established as Equation (4):

Rough grinding (Fine grinding/Ultra-fine grinding):

(4) $$\{\begin{array}{c} \min(Ra(x),T_{G}(x),-T_{D}(x))\\ s.t.\;500\leq n_{g}\leq 4000\\ \;\;0.1\leq T_{G}\leq 1.0\\ \;\;0.1\leq T_{D}\leq 1.0\\ \;\;Ra\leq 2(1.2/0.05) \end{array}$$

As one of the evaluation criteria of the grinding machine tool, the surface roughness is constrained to reduce the dimension of the multi-objective programming mathematical model.

Rough grinding (Fine grinding/Ultra-fine grinding) (Equation (5)):

(5) $$\{\begin{array}{c} \max(T_{D}(x)-T_{G}(x)=110.741-0.0059n_{g}-27.4557v_{h}-29.6937v_{v})\\ \begin{array}{cc} s.t. & \;\;\;\;\;\;\;\;500\leq n_{g}\leq 4000 \end{array}\\ \;\;0.1\leq v_{h}\leq 1.0\\ \;\;0.1\leq v_{v}\leq 1.0\\ Ra=-0.2717+{0.0004n}_{g}+0.2668v_{h}+0.4739v_{v}=2(1.2/0.05) \end{array}$$

To solve this optimization equation, we convert this multi-objective programming into a standard form (Equation (6)):

(6) $$\{\begin{array}{c} \max(110.741(89.0334/105.996)-0.0059n_{g}-27.4557v_{h}-29.6937v_{h})\\ \begin{array}{c} s.t.-667v_{h}-1184.75v_{h}+x_{1}=-1769.25(667v_{h}+1184.75v_{h}+x_{1}=3179.25/304.25)\\ -v_{h}+x_{2}=-0.1 \end{array}\\ \begin{array}{c} v_{h}+x_{3}=1\\ -v_{v}+x_{4}=-0.1 \end{array}\\ \begin{array}{c} v_{v}+x_{5}=1\\ x_{1},x_{2},x_{3},x_{4},x_{5}\geq 0 \end{array} \end{array}$$

where *x*_1_, *x*_2_, *x*_3_, *x*_4_, and *x*_5_ are slack variables. 

### 4.3. Optimization Results

The solution results via the dual simplex method are shown in Table 9. BO and AO represent ‘before optimization’ and ‘after optimization’, respectively. The optimized grinding parameters and indicators are compared with the traditional indicators.

The optimization results are shown in the table above. Under the premise of the same surface roughness, the grinding wheel life is extended, and the grinding time is shortened. Specifically, for rough grinding, the grinding time acquired is reduced by 0.729 min, and the grinding wheel life is increased by 14.6682 h. For fine grinding, the grinding time acquired is reduced by 1.9437 min, and the grinding wheel life is increased by 8.5464 h. For ultra-fine grinding, the grinding time acquired is reduced by 1.6453 min, and the grinding wheel life is increased by 5.7238 h. For the total grinding process including rough grinding, fine grinding, and ultra-fine grinding, the time acquired is reduced by 4.3174 min, and the grinding wheel life is increased by 28.9384 h. The optimization results are validated on the experiment grinding machine tool YKZ7230.

## 5. Discussion

To optimize and improve the grinding process of 20CrMnTiH gear, firstly, we applied the pixel method to analyze the worm wheel grinding process, and the analysis results show that similar to the traditional cutting process, three grinding parameters—the feed rate, the grinding wheel speed, and the grinding wheel frame moving speed—have a comprehensive impact on the grinding indicators, which is decided by the worm wheel grinding principle and characteristics [24]. Secondly, 28 groups of variable controlled experiments were carried out, and the experiment results indicate that the impact of the grinding parameters on the grinding indicators is linearly correlated. Finally, through the multi-objective optimization of grinding, the time required to achieve the same accuracy is greatly shortened, and the grinding wheel life is improved significantly, which indicates that the ceramic micrystalline grinding wheel is used more fully and effectively.

## 6. Conclusions

Based on the ceramic microcrystalline corundum grinding wheel grinding 20CrMnTiH gear workpiece, a large number of variable controlled group experiments were conducted. The study focuses on the impact and optimization analysis of grinding parameters on the grinding indicators. In the course of this analysis, we have demonstrated:

1. The grinding parameters are linearly correlated to the grinding indicators;
2. The precision, efficiency, and economy of the grinding process are improved comprehensively via the comprehensive optimization of the grinding parameters.

## Figures and Tables

**Table 1:** Chemical composition of microcrystalline corundum.

Component	Al_2_O_3_	Na_2_O	Si_2_O	Magnetic Substance
**Content (weight %)**	99.0	0.4	0.03	0.05

**Table 2:** Physical properties of microcrystalline corundum.

Hardness (kg/mm^2^)	Granularity (μm)	Bulk Density (g/cm^3^)	Linear Elastic Coefficient (1/K)	Compressive Strength (MPa)
99.6	80	3.6	8 × 10^−6^	340

**Table 3:** Chemical composition of 20CrMnTiH.

Component	C	Si	Mn	Cr	S	P	Ni	Cu	Ti
**Content (weight %)**	0.18	0.26	0.9	1.2	0.02	0.02	0.3	0.25	0.08

**Table 4:** Physical properties of 20CrMnTiH.

Hardness (HB/HRC)	Yield Strength (MPa)	Tensile Strength (MPa)	Elongation (%)	Shrinkage (%)	Elastic Modulus (GPa)	Density (kg/m^3^)
217/60	835	1080	10	45	207	7.8 × 10^3^

**Table 5:** The parameters of 20CrMnTiH gear workpieces.

Modulus	Number of Teeth z	Pressure Angle α	Helix Angle β	Width (B/mm)
4	70	20°	15°	30

**Table 6:** The experiment grouping and results of exploring the impact of the grinding wheel speed.

Group No.	Feed Rate *v_h_* (mm/min)	Grinding Wheel Frame Speed *n_g_* (mm/rpm)	Grinding Wheel Speed *v_v_* (rpm)	Surface Roughness Ra (μm)	The Time Required to Achieve Machining Accuracy *T_G_* (min)	Grinding Wheel Life *T_D_* (min)
1	0.3	0.6	500	0.098	15.3	96.2
2			1000	0.265	11.2	92.4
3			1500	0.486	9.6	88.7
4			2000	0.862	8.2	84.3
5			2500	1.206	7.6	76.8
6			3000	1.326	6.8	68.2
7			3500	1.465	5.6	60.5
8			4000	1.523	4.2	52.3

**Table 7:** The experiment grouping and results of exploring the impact of the feed rate.

Group No.	Feed Rate *v_h_* (mm/min)	Grinding Wheel Frame Speed *n_g_* (mm/rpm)	Grinding Wheel Speed *v_v_* (rpm)	Surface Roughness Ra (μm)	The Time Required to Achieve Machining Accuracy *T_G_* (min)	Grinding Wheel Life *T_D_* (min)
1	0.1	0.6	3000	0.268	10.2	91.6
2	0.2			0.354	9.8	93.4
3	0.3			0.423	9.6	98.2
4	0.4			0.782	8.2	92.6
5	0.5			0.965	7.0	85.4
6	0.6			1.135	6.2	76.3
7	0.7			1.212	5.4	70.8
8	0.8			1.298	4.8	62.5
9	0.9			1.365	4.4	56.9
10	1.0			1.411	4.2	45.6

**Table 8:** The experiment grouping and results of exploring the impact of the grinding wheel frame speed.

Group No.	Feed Rate *v_h_* (mm/min)	Grinding Wheel Frame Speed *n_g_* (mm/rpm)	Grinding Wheel Speed *v_v_* (rpm)	Surface Roughness Ra (μm)	The Time Required to Achieve Machining Accuracy *T_G_* (min)	Grinding Wheel Life *T_D_* (min)
1	0.3	0.1	3000	1.106	11.5	97.2
2		0.2		1.165	11.1	92.6
3		0.3		1.267	10.6	90.2
4		0.4		1.301	9.8	85.6
5		0.5		1.325	9.2	82.7
6		0.6		1.354	8.6	80.5
7		0.7		1.402	8.0	77.9
8		0.8		1.453	7.1	73.5
9		0.9		1.462	6.2	68.7
10		1.0		1.485	5.4	64.6

**Table 9:** The solution results via the dual simplex method. BO: before optimization; AO: after optimization.

Group No.	Rough Grinding BO	Rough Grinding AO	Fine Grinding BO	Fine Grinding AO	Ultra-Fine Grinding BO	Ultra-Fine Grinding AO
*v_h_* (mm/min)	1	0.8763	0.3	0.1	0.15	0.1
*v_v_* (mm/rpm)	2	1	0.5	0.1	0.2	0.1
*n_g_* (rpm)	3800	4000.0079	1800	2427.8	500	619.075
Ra (μm)	2	2	1.2	1.2	0.05	0.05
*T_G_* (min)	4.5	3.771	20	18.0563	23.5	21.8547
*T_D_* (h)	22.5	37.1682	100	108.5464	117.5	123.2238
